# Reproduction in Animal Models of Lysosomal Storage Diseases: A Scoping Review

**DOI:** 10.3389/fmolb.2021.773384

**Published:** 2021-11-12

**Authors:** Daniela Vuolo, Cinthia Castro Do Nascimento, Vânia D’Almeida

**Affiliations:** ^1^ Department of Pediatrics, Universidade Federal de São Paulo, São Paulo, Brazil; ^2^ Department of Biosciences, Universidade Federal de São Paulo, Santos, Brazil; ^3^ Department of Psychobiology, Universidade Federal de São Paulo, São Paulo, Brazil

**Keywords:** lysosomal storage diseases, mucopolysaccharidosis, sphingolipidosis, lipidosis, reproduction, sperm, testis, ovary

## Abstract

**Background:** Lysosomal storage diseases (LSDs) are caused by a mutation in a specific gene. Enzymatic dysfunction results in a progressive storage of substrates that gradually affects lysosomal, cellular and tissue physiology. Their pathophysiological consequences vary according to the nature of the stored substrate, making LSDs complex and multisystemic diseases. Some LSDs result in near normal life expectancies, and advances in treatments mean that more people reach the age to have children, so considering the effects of LSDs on fertility and the risks associated with having children is of growing importance.

**Objectives:** As there is a lack of clinical studies describing the effect of LSDs on the physiology of reproductivity, we undertook a scoping review of studies using animal models of LSDs focusing on reproductive parameters.

**Methods:** We searched six databases: MEDLINE, LILACS, Scopus, Web of Science, Embase and SciELO, and identified 49 articles that met our inclusion criteria.

**Results:** The majority of the studies used male animal models, and a number reported severe morphological and physiological damage in gametes and gonads in models of sphingolipidoses. Models of other LSDs, such as mucopolysaccharidoses, presented important morphological damage.

**Conclusion:** Many of the models found alterations in reproductive systems. Any signs of subfertility or morphological damage in animal models are important, particularly in rodents which are extremely fertile, and may have implications for individuals with LSDs. We suggest the use of more female animal models to better understand the physiopathology of the diseases, and the use of clinical case studies to further explore the risks of individuals with LSDs having children.

## Background

Inborn errors of metabolism are disorders caused by genetic mutations which result in enzymatic defects that interrupt specific metabolic pathways and interfere in the synthesis, degradation, storage or transport of molecules (Scriver et al., 2001). More than 750 inborn errors of metabolism have been described to date and, affecting approximately 1:1,000 births (Saudubray and Garcia-Cazorla, 2018).

Lysosomal storage diseases (LSDs) are a specific category of inborn errors of metabolism caused by gene mutations that affect the activity of lysosomal hydrolases. Consequently, substrates are continuously accumulated inside the lysosomes and in interstitial spaces, according to the particularity of each disorder (Parkinson-Lawrence et al., 2010; Platt et al., 2018). They are genetically and clinically heterogeneous diseases, affect multiple organs and tissues and are progressive. In some cases, patients present an attenuated phenotype, while in other cases, they present a severe manifestation. The reason for this heterogeneity is still unclear and discordant between authors.

LSDs are divided into subcategories according to the biochemical nature of the storage substrate: sphingolipidoses, mucopolysaccharidoses, glycogenoses, glycoproteinoses and lipid storage diseases (Parkinson-Lawrence et al., 2010). There are also some disorders that are caused by post-translational modification defects on enzymes, disorders in integral protein membranes and endoplasmic reticulum proteins that interfere in lysosomal and cellular metabolism (Platt et al., 2018).

The diagnosis of LSDs is based on clinical symptoms, an analysis of enzymatic activity in blood, an analysis of substrates in urine and, gene sequencing (Parenti et al., 2015; Platt et al., 2018). To date, enzyme replacement therapy (ERT) is the most established treatment for some LSDs, such as Gaucher, Fabry, Pompe, and Wolman diseases, α-mannosidosis and mucopolysaccharidoses (MPS) type I, II, IV, VI and VII (Parenti et al., 2013; Platt et al., 2018).

Animal models have been used in a wide range of studies on LSDs. They are particularly important in clarifying the pathophysiology of the different diseases and also in exploring therapeutic options (Suzuki et al., 2003). A few of these studies have also looked at the effects of LSDs on various aspects of fertility and reproduction. This is an important topic and an area that requires greater attention given the fact that some LSDs result in near-normal life expectancies, and that more effective treatments are responsible for a greater number of people with the disease reaching an age to consider having children (Platt et al., 2018; Marques and Saftig, 2019).

Papaxanthos-Roche et al. (2019) carried out a study to analyze the characteristics of the semen and genital tract of male patients with Fabry disease and concluded that 52.9% of the patients had at least one semen abnormality; the most common changes included a reduced sperm count, followed by reduced semen volume and sperm vitality. However, a study by Hauser et al. (2005) found that the plasmatic sexual hormonal profiles of 13 patients (6 women and 7 men) were normal.

Stewart et al. (2016) described cases of eight mothers and five fathers with different types of MPS and reported that the women had high-risk pregnancies, but, with appropriate monitoring, all babies developed normally and the mothers suffered no adverse effects, while pregnancies from fathers with MPS were uncomplicated; The children were also healthy, with normal growth and development. Another case report with MPS I revealed that ERT was safe for the mother during pregnancy and for her baby (Castorina et al., 2015), while four successful pregnancies were described in MPS I women treated by bone marrow transplantation (Rémerand et al., 2009). On the other hand, signs of precocious puberty were detected in some boys affected with MPS I and MPS III (Milazzo et al., 2014) and MPS III A (Tylki-Szymanska & Metera).

Other studies with women affected with MPSs revealed high-risk pregnancies and deliveries (Bacchus and Peterson, 1980; Anbu et al., 2006; Delgado et al., 2015). Wilson et al. (2018) highlighted the importance of MPS patients knowing the risks and having genetic counseling. Sechi et al. (2013) evaluated 32 women affected by glycogenosis type I and although many of them presented delayed menarche, irregular cycles, and polycystic ovaries, spontaneous pregnancies were recorded in some patients.

Despite these results, there is still a significant lack of knowledge about this wide category of disorders and fertility. The present study, therefore, aimed to analyze their influence on reproductive parameters in the available animal models, through a scoping review.

## Methods

A scoping review was chosen as the method for the present study as LSDs are a heterogenous category of disorders with each available study focusing on different diseases, grades of disease progression and tissues, and some of them including the results of therapeutic interventions in their observations. For this reason, we decided to evaluate all the published material related to this theme following a systematic search which included both male and female reproductive parameters in animal models of LSDs to provide a descriptive view of this subject.

### Data Collection

To carry out a comprehensive search, the following databases were selected: MEDLINE, Scopus, Web of Science, LILACS, SciELO and Embase (via *Portal de Periódicos CAPES,* a Brazilian Institutional databay). Data collection was carried out for studies published until July 2021. The following keywords and Medical Subject Headings (MeSH) terms were used: (mucopolysaccharidosis OR sphingolipidosis OR glycogenosis OR glucoproteinosis OR “multiple sulphatase deficiency” OR lipidosis OR ″i cell disease” OR mucolipidosis OR gangliosidosis OR lipofuscinosis OR fucosidosis OR fabry OR gaucher OR “niemann pick” OR pompe OR “lysosomal storage”) AND (mice OR rat OR cat OR dog) AND (testis OR epididymis OR sperm OR spermatozoa OR semen OR acrosome OR gamete OR prostate OR “seminal vesicle” OR ovary OR uterus OR oocyte OR “pellucid zone” OR fertility OR sexual).

It was necessary to limit the search period to 2010 to 2021 in three databases: Web of Science, Scopus and Embase, due to the large number of results returned that would have made our search unfeasible. No initial time limit was used in the search of the other databases: MEDLINE, LILACS and SciELO.

We selected all abstracts according to the following inclusion criteria: 1. studies involving an animal model of LSD that mentioned or were directly focused on male or female reproductive parameters; 2. studies involving substrate storage in gonads that did not necessarily represent a specific LSD, but are lysosomal related disorders and would help in understanding the effects of such unsuitable accumulation in these tissues. 3. Studies directly focused on reproductive parameters of LSD models. All abstracts were analyzed independently by two of the authors and, in the case of any disagreement, the third author decided the inclusion or exclusion.

## Results and Discussion

**FIGURE 1:**
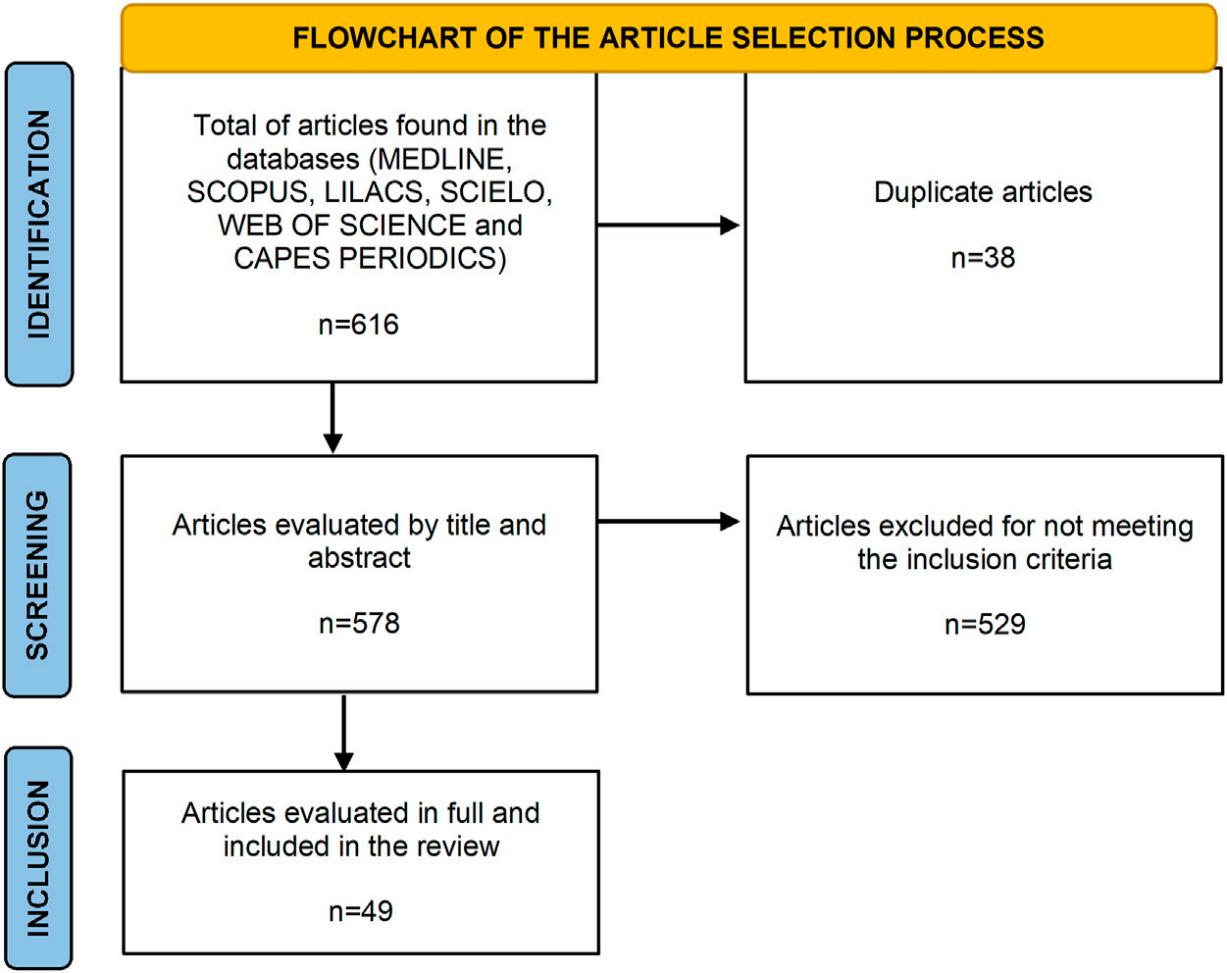
Flowchart of identification, screening and inclusion studies found by the database.

The initial database search resulted in a total of 616 articles: 268 in Embase, 252 in MEDLINE, 50 in Web of Science, 40 in Scopus, 5 in SciELO and 1 in LILACS. From these, 38 duplicate were excluded; From the remaining 578, 49 met the inclusion criteria, and the details are given in Figure 1.

The first article involving animal models and reproductive parameters included in this review was published in 1989, followed by publications in the 1990s and 2000s, until 2021, with the largest number of studies (five) being published in 1999 and 2006. The highest concentration of studies by disease category were those relating to MPS I (7), followed by NPC1 (6) and NPC (3). The list of all diseases or enzymes found in our search is detailed in Table 1. We separated the 49 studies identified according to the disease or the most important findings, as we describe above.

**TABLE 1:** List of diseases or enzymes studied by disease category, and the related studies.

disease category	Disease	Enzyme	Authors	Reproductive damage
SPHINGOLIPIDOSES		Sulfogalactosylglycerolipid	Tanphaichitr et al. (2018)	Spermatogenesis; Sertoli cell; Testicle
			Kongmanas et al. (2021)	
		Sulfated glycoprotein-1	Hermo and Andonian (2003)	Epididymis
	Fabry	a-galactosidase A	Murray et al. (2007)	Testicle; Testosterone
			Shen et al. (2015)	
	Krabbe	Galactosylceramidase	Luddi et al. (2005)	Sperm; Testicle; Epididymis; Ovary; Uterus; Spermatogenesis
			Hu et al. (2012)	
			Piomboni et al. (2014)	
	Niemann Pick A	Acid sphingomyelinase	Butler et al. (2007)	Sperm
	Niemann Pick B			
	Tay-sachs	Hexosaminidase A	Hermo et al. (1997)	Testicle; Epididymis; Efferent ducts
			Trasler et al. (1998)	
			Adamali et al. (1999a) ^12^	
			Seyrantepe et al. (2018)	
	Sandhoff	Hexosaminidase B	Hermo et al. (1997)	Testicle; Epididymis; Efferent ducts; Fertility
			Trasler et al. (1998)	
			Adamali et al. (1999b) ^1^	
			Juneja (2002)	
	Gaucher	B-glycosidase 1	Korschen et al. (2013)	Testicle
		B-glycosidase 2	Yildiz et al. (2006)	Testicle; Sperm; Sertoli cell
			Korschen et al. (2013)	
		Saposin C	Morales et al. (2000)^2^	Testicle; Epididymis; Prostate; Seminal vesicle
	Metachromatic leukodystrophy	Arylsulfatase A		Sperm; Sertoli cell; Spermatogenesis
			Wu et al. (2019)	
			Xu et al. (2011)	
		Prosaposin	Morales et al. (2000a) ^1^	Prostate; Testicle; Seminal vesicle; Testosterone
		Saposin B	Morales et al. (2000b) ^2^	Testicle; Epididymis; Prostate; Seminal vesicle
Mucopolysaccharidoses	MPS I	α-L-iduronidase		Testicle; Ovary; Sexual behavior; Sperm; Epididymis; Seminal vesicle; Prostate; Sertoli cell
			Chung et al. (2007)	
			Schneider et al. (2016)	
			do Nascimento et al. (2019)^12^	
			do Nascimento et al. (2019)	
			do Nascimento et al. (2020)	
			Barbosa Mendes et al. (2019)	
			do Nascimento et al. (2014)	
	MPS II	Iduronate-2-Sulfatase	Higuchi et al. (2012)	Testicle
	MPS VII	β-glucuronidase	Soper et al. (1999)	Fertility; Sexual behavior
Glycoproteinoses	A-mannosidosis	A-lysosomal mannosidase	Stinchi et al. (1999)	Testicle
	B-mannosidosis	B-mannosidase	Zhu et al. (2006)	Epididymis
	Galactosialidosis	Cathepsin A	Hu et al. (2012)	Epididymis; Testicle; Ovary; Uterus
	Fucocidosis	α-1-Fucosidase	Taylor et al. (1989)	Testicle; Epididymis; Prostate; Sperm; Sertoli cell
			Veeramachaneni et al. (1998)	Efferent ducts
Integral Membrane Protein Disorders	Niemann Pick C	Acid sphingomyelinase	Roff et al. (1992), Roff et al. (1993)	Hormonal expression; Testicle; prostate; Epididymis; Seminal vesicle; Sperm
			Butler et al. (2002)	
	Niemann Pick C1	NPC 1 and 2 intracellular cholesterol transporter	Gévry and Murphy (2002)	Steroid hormones; Fertility; Ovary; Testicle; Sperm
			Erickson et al. (2002)	
			Gévry et al. (2004)	
			Xie et al. (2006)	
			Fan et al. (2006)	
			Donohue et al. (2009)	
	Niemann Pick C2		Busso et al. (2010), Busso et al. (2014)	Sperm; Epididymis; Fertility; Ovulation
	Mucolipidosis IV	Mucolipin 1	Wang et al. (2019)	Ovary; Luteal cells
Neuronal Ceroid Lipofuscinosis	JNCL		Staropoli et al. (2012)	Epididymis
	CLN10 (CTSD)	Cathepsin D	Hermo and Andonian (2003)	Epididymis
Glycogen Storage Disease	Pompe	Lysosomal A-glucosidase	Ponce et al. (1999)	Testicle; Efferent ducts; Epididymis
Lipidoses	Lipidosis		Geist and Lullmann-Rauch (1994)	Ovary; Uterus

CLN; neuronal ceroid-lipofuscinosis, MPS; Mucopolysaccharidosis, JNCL; juvenile neuronal ceroid lipofuscinosis.

Studies specifically focused on female models were rare in our search, probably due to their cyclic hormonal variations, which result in difficult experimental conditions. From the 49 included studies, only 6 were exclusively related to female models.

Studies involving knockout models generally use approximately five animals per group. The crossings are usually performed with heterozygotes and only 25% of the littermates are knockout (males and females). The animals are fragile and it is common to lose some when investigating an advanced time point of a disease, because they die before reaching the established age. Thus, the sample size is a common limitation in this category of study. The majority of works used approximately 5 animals per group, with a of 2 animals/group in a few studies. Studies using more than 10 animals were rare.

### Studies With LSDs Models That Mention the Genital System

Some studies were focused on a broad characterization of knockout mice and mentioned the gonads as one of the objects of analysis. Seyrantepe et al. (2018) examined a model of Tay Sachs disease using a double mutant mouse (*Hexa*−/− and *Neu3*−/−), since it is known that sialidase (Neu3) is alternatively used to degrade the ganglioside GM2 in the absence of hexosaminidase A (Hex A). This ganglioside is known to be stored in neurons and macrophages, but testicular samples were analyzed by electron microscopy and Sertoli cells were found to be filled with lamellar bodies, suggesting lipidic storage in 4.5-month-old mice. Staropoli et al. (2012) detected numerous vacuoles in the epididymal cells in a mouse model of juvenile neuronal ceroid lipofuscinosis (JNCL). A *Cln3* gene knock-out model was used, as mutations in this gene are the most frequent causes of JNCL. Cognitive impairments and vacuolated lymphocytes were expected, but after a broad phenotyping study, the epididymis was one of the organs with greater lipid storage.

Murray et al. (2007) evaluated a mouse model of Fabry disease, deficient in producing α-galactosidase A, a lysosomal enzyme that degrades glycosphingolipids, particularly globotriaosylceramide (Gb3). Mutant mice submitted to intravenous enzyme replacement therapy had their tissue evaluated using immunostaining. This showed that the enzymatic infusion had heterogenous systemic distribution, since the antibody was detected in the liver, kidneys, heart, adrenal gland, spleen, bone marrow and also in the testes.

A number of models of oligosaccharidosis presented signs of damage in reproductive tissues. Stinchi et al. (1999) generated a knockout model of α-mannosidosis, a lysosomal enzyme that degrades asparagine-linked carbohydrate cores of glycoproteins. Morphological signs of storage were found in the liver, kidneys, spleen, brain and testes. Similarly, Zhu et al. (2006) studied a knockout model of β-mannosidosis. The model failed to cleave a mannose sugar, present in oligosaccharides and glycoproteins. Among other findings, under optic and electronic microscopy the epididymis was seen to be filled with vacuoles.

Higuchi et al. (2012) evaluated a mouse model of mucopolysaccharidosis type II (MPS II) with animals submitted to intraventricular enzyme replacement therapy. The enzyme degrades glycosaminoglycans (GAGs) present in the intracellular and extracellular environment. Treated MPS II mice presented less storage in many tissues, including in the testes and ovaries. The authors had expected an increase in recombinant enzyme activity in a brain-specific manner; however, contrary to their expectations, the enzyme activity was also high in some peripheral tissues.

### Studies Specifically Related to Reproductive Parameters

#### Mucopolysaccharidoses

Among MPSs models, MPS I is the most investigated type of the disease in respect of reproductive parameters. Testicular GAG deposits were first described by Chung et al. (2007). The researchers administrated a retroviral vector containing the gene of α-L-iduronidase in neonates in an MPS I model. The clearance of GAGs was improved in many organs, including the testes, in comparison to untreated mice. This was one of the first mentions of gonads in an MPS I model. A similar study was performed by Schneider et al. (2016), but involved intravenous enzyme replacement therapy. In this study, researchers evaluated different tissues of wild type mice, untreated MPS I mice, MPS I continuously treated since the neonatal phase and, MPS I treated mice with an interruption of 2 months (from 2 until 4-months-old). Among the analyzed tissues, the testes of 6-month-old treated mice had a preserved morphology (even with the therapeutic interruption) while untreated mice presented numerous vacuoles in the interstitial compartment.

Specific studies focused on male reproduction in models of MPS I were performed from 2014 to 2020. In 2014, do Nascimento et al. detected lower sperm production in 6-month-old *Idua*−/− mice and histologic changes in the seminiferous tubules and interstitial compartment. The same group performed a more detailed investigation and found that under the same grade of disease progression, sperm were morphologically normal and motile despite the evident morphological changes found in epididymis, prostates and seminal vesicles (do Nascimento et al., 2019a; do Nascimento et al., 2019b). Although 3 and 6-month-old males presented signs of motor limitations, they were able to copulate and impregnate females. (Barbosa Mendes et al., 2019; do Nascimento et al., 2019a). Under ultrastructure examination, numerous vacuoles were found in interstitial compartments, myoid cells and some lamellar bodies in Leydig cells of 6-month-old mice (do Nascimento et al., 2019a). An important sign was detected in Sertoli cells which suggested incomplete digestion of substrates, since vesicles similar to autophagosomes, autolysosomes and lysosomes were detected in a different proportion between the wild type and *Idua*−/− mice (do Nascimento et al., 2019b). This data is important because Sertoli cells have a great demand in respect of cellular digestion during spermatogenesis (Xu et al., 2011).

It is important to clarify that a single deficient enzyme that causes the accumulation of a specific substrate may lead to secondary storage as a consequence of the unbalance of the metabolic pathways. In MPS I and MPS III models for example, secondary storage of cholesterol and glycosphingolipids in neurons has been detected, even in an LSD that primarily involves the storage of GAGs (McGlynn et al., 2004; Walkley, 2004; Walkley et al., 2005). This secondary lipid storage must also indirectly affect reproductive functions and may even explain some of the damage found in MPS models.

Three-month-old females in an MPS I model were capable of exerting sexual behavior, regardless of their motor impairments (Barbosa Mendes et al., 2019). In the same study, it was demonstrated that plasma steroid hormonal levels (testosterone, progesterone and 17-β-estradiol) do not differ between wild type and *Idua−/−* mice, both in males and females.

Another study related to sexual behavior and fertility of MPSs was performed in 1999 by Soper et al., using males and females in an MPS VII model. A group of animals were intravenously treated with the recombinant enzyme β-glucuronidase. The therapy significantly reduced GAG storage in most tissues, increased life span and improved the animals’ cognitive ability and mobility. Treated MPS VII mice were able to copulate and generate pups with a better efficiency in comparison to untreated ones. The ovaries of young adult MPS VII mice had follicles and corpora lutea, and the testes of treated males generated sperm. The authors suggested that the reproductive failure in MPS VII mice is related to impaired mobility and cognition, and the enzyme replacement restores mating capacity. Additionally, postnatal losses were more extreme when the mother was untreated for MPS VII, though slightly less than treated mothers.

### Sphingolipidoses

The biosynthesis, degradation and transport of lipids are extremely important to steroidogenesis (Xie et al., 2006). Steroid hormones, such as testosterone, progesterone and 17-β estradiol, use cholesterol as a precursor molecule. All these hormones control gametogenesis, estrous cycles, the maintenance of gonads, sexual behavior, parental care and the manifestation of male and female secondary sexual characters (Hull et al., 2007). For this reason, the worst morphological and physiological damage was present in models of lipidoses, sphingolipidoses or ceroid lipofuscinoses.

Sphingomyelinases degrade sphingomyelin into ceramide and phosphorylcholine. The pathophysiology in Niemann Pick disease is primarily due to the accumulation of sphingomyelin and other metabolically related lipids within the cells and tissues (Levade et al., 1999).

Butler et al. (2002) used a knockout model of Niemann-Pick A and B, an acid sphingomyelinase deficient mouse (ASM). Testicular sections of ASM deficient mice presented vacuoles and Sertoli cells full of vesicles with undigested material. Sperm presented changes in their plasma membrane, in acrosome reaction and failed in the mitochondrial membrane polarization. Morphological damage in the sperm head and flagellum were also noted, and the epididymal epithelia were filled with vacuoles. Despite all these structural problems, males were capable of mating but litter size and the number of litters were decreased compared to wild types. They also evaluated crossings with female mutants and litters were even more prejudiced, with a lower number of litters and litter sizes (Butler et al., 2002).

The same authors examined knockout and heterozygotes of the ASM model and detected two different sperm populations from *Asm+/−* (normal and affected sperm). They performed *in vitro* fertilization and could select normal sperm based on their morphology and on their mitochondrial membrane potential. The authors affirmed that sperm sorting offers several advantages over the existing assisted reproduction options for Niemann-Pick disease carrier couples and could have a major impact on the prevention of this, and perhaps other, genetic diseases (Butler et al., 2007).

Adamali et al. (1999a) studied the relevance of hexosaminidase (Hex) in the testes and epididymis. This lysosomal enzyme exists as two isoenzymes (Hex A, (subunit αβ and Hex B, subunit ββ) that degrade gangliosides (GM2), and both isoenzymes and GM2 are abundant in neurons and present in other visceral organs such as the liver and kidneys. The disruption of the *Hexb* gene, that encodes subunit β, impairs the activity of both Hex A and Hex B and mimics Sandhoff disease, a severe neurodegenerative LSD. In the testis, seminiferous tubules were similar to wild types, Sertoli and germ cells appeared normal, but myoid and interstitial macrophages showed an increased number of lysosomes. The epithelial cells of efferent ducts and epididymal ducts also had numerous lysosomes in 1-month-old affected male mice. The same group was examined in a Tay Sachs model (knockout for the subunit α, which disrupts only Hex A). In this model, the initial segments of the epididymal duct presented more evidence of lysosomal storage than the other segments (Adamali et al., 1999b). Both studies showed the importance of Hex in the biosynthesis and degradation of gangliosides in testicular and epididymal ducts.

Trasler et al. (1998) also used the mouse model of Sandhoff disease and found that testes weight, morphology and sperm count were unaffected in knockout mice. Epithelial cells of the epididymal and efferent ducts were affected by extensive lysosomal abnormalities. In contrast to the brain, where GM2 ganglioside accumulates, mutant mice accumulated two non-GM2 gangliosides in the epididymis. The mice were fertile, but their litter size was reduced after 9 weeks of the disease progression. The authors suggested that testes derived glycolipids could not be degraded and accumulated in lysosomes, leading to epididymal dysfunction and abnormalities in the epididymal luminal environment that supports sperm maturation.

Juneja. (2002) evaluated the fertility profile of male and female *Hexb−/−* mice. Males and females were fertile up to approximately 2.5-months-old and 2-months-old respectively. Knockout males presented a reduction to an absence in mating behavior from 84 to 94-days-old (almost 3-months of disease progression). Sperm from *Hexb−/−* showed lower *in vitro* fertilization. Three-month-old *Hexb−/−* females in contact with healthy males were unable to be impregnated, regardless of the presence of a vaginal plug. However, oocytes from *Hexb−/−* could be fertilized at a lower efficiency by spermatozoa from wild type mice. Hermo et al. (1997) detected a high expression of β-hexosaminidase in the epididymis and testes compared to other tissues and highlighted its relevance to the production and maturation of sperm.

Luddi et al. (2005) studied a male mouse model of globoid cell leukodystrophy (Krabbe disease), using mice with a mutation in the galactosylceramidase (GALC) gene. GALC hydrolyzes galactose from galactosylceramide, a typical component of the myelin membrane. The undigested substrate for GALC, galactosyl-alkyl-acyl-glycerol (GalAAG), accumulates in the testes and affects sperm number, maturation, morphology and function. The study proved that GALC plays a critical role in spermiogenesis. Another study using the same model confirmed the sperm and testicular morphological abnormalities and detected interference in the hypothalamus-pituitary-gonadal axis under RT PCR analysis, since LH and FSH were significantly decreased in mutant mice (Piomboni et al., 2014).

Wu et al. (2019) evaluated a model of metachromatic leukodystrophy that used arylsulfatase A (ARSA) knockout mice; ARSA catalyzes glycolipids of myelin. In this study, the researchers found that this enzyme is present in the acrosomal vesicle and reacts with cumulus oocyte complexes. Sperm from ARSA null mice showed a significant delay in the dispersion of cells present in cumulus oocyte complexes, which interfere in the efficiency of fertilization. The same model was used by Xu et al. (2011) who demonstrated that Sertoli cells depend on ARSA to degrade sulfogalactosylglycerolipid (SGG), the major sulfoglycolipid of sperm that is directly involved in the cell adhesion between testicular germ cells and Sertoli cells.

During spermatogenesis, approximately 50% of germ cells are sent to apoptosis to avoid becoming abnormal sperm. Sertoli cells phagocyte these apoptotic germ cells and degrade part of the cytoplasm of sperm during spermiogenesis. *Arsa−/−* mice had SGG accumulated in Sertoli cells, with lysosome swelling, impaired spermatogenesis and lower fecundity *in vitro* and *in vivo*, when males were older than 5 months (Xu et al., 2011). Additionally, increased levels of superoxide and hydrogen peroxide were found in Sertoli cells of *Arsa−/−* mice, which may explain the decrease in spermatogenesis and increased abnormal sperm population in this knockout model (Kongmanas et al., 2021). Tanphaichitr et al., 2018 highlighted the importance of SGG on male reproduction. Knockout mice for *Cgt* and *Cst*, two enzymes that act on SGG biosynthesis, also had their spermatogenesis disrupted.

Morales CR. et al. (2000), Morales C. R. et al. (2000) evaluated some male reproductive parameters in a mouse deficient of prosaposin, an enzyme that degrades sphingolipids. Prosaposin is targeted to lysosomes and processed into smaller molecules (saposins A, B, C, and D). The deficiency of saposin B and C results in metachromatic leukodystrophy and Gaucher diseases respectively. The knockout mouse presented small testes, epididymis, seminal vesicles and prostates; prostatic secretory cells were absent; and spermiogenesis was reduced. However, plasma testosterone was higher in knockout males compared to wild types. Prostate sections immunostained with antiandrogen receptor antibody were similar between groups, but the MAPK pathway was inactive in the mutant mice.

Körschen et al. (2013) worked with a GBA 2 knockout mouse. GBA 1 and GBA 2 are glucosidases that cleave glucosylceramide to glucose and ceramide. Mutation in GBA1 causes Gaucher disease. However, GBA 2 is localized in the endoplasmic reticulum and Golgi and has a strong association with cellular membranes. Studying GBA 2 may elucidate the pathophysiology of Gaucher disease. Its absence results in the storage of glucosylceramide in many tissues, including the testes, and impairs sperm development. Sperm with large round heads, abnormal acrosomes and defective motility were detected by Yildiz et al. (2006) using the same model.

Shen et al. (2015) detected increased androgen receptor (AR) signaling in a mouse model of Fabry disease with deficient α-galactosidase, which catabolizes glycosphingolipids. The blocking of AR signaling by castration or by treatment with an AR antagonist prevented cardiac and kidney hypertrophy, two common clinical manifestations of patients affected by Fabry disease. They concluded that the AR pathway interferes in the pathogenesis of the disease and suggested the blocking of this signaling as a novel therapeutic approach.

### Integral Membrane Protein Disorders

Some diseases are not directly related to a poor lysosomal enzymatic activity, but to a failure in the transport of some substrates or ions from cytoplasm to lysosomes and from lysosomes to cytoplasm. Some integral proteins transport lipid substrates to lysosomes and some ion channels regulate the lysosomal ionic environment. This molecular traffic is essential to regulate the cell signaling from cytoplasm to nucleus and also the intra-lysosomal metabolic pathways (Medina and Ballabio, 2015; Platt et al., 2018).

Erickson et al. (2002) evaluated a double mutant mouse (*Npc−/−* and *Mdr−/−*) model. This *Npc*−/− mouse mimics Niemmann-Pick Type C disease, a progressive neurological disease that interfere in cholesterol transport, not specifically in the activity of a lysosomal hydrolase. Despite normal folliculogenesis and normal progesterone levels, *Npc*−/− female mice were infertile and presented a lack of implantation because of abnormal cellular cholesterol homeostasis. However, *Npc*−/− and *Mdr*−/− double mutant females had their fertility recovered, as the *Mdr* gene codes a multiple drug resistance (MDR) P-glycoprotein, a plasma membrane protein implicated in the movement of drugs and lipids across membranes. While the neurological disease continued at its usual rate, preventing the females from taking care of their litters, double mutant females became fertile, which demonstrated the participation of a system of proteins in the control of cellular cholesterol transport.

Donohue et al. (2009) also evaluated a female model of *Npc−/−*, but in this specific model, fibrillary astrocytes expressed NPC1 protein, using glial fibrillary acidic protein (GFAP) promoter. This selective expression of NPC1 corrected sterility of *Npc*−/− females as a result of restoring hypothalamic control of the pituitary. These results were reinforced by Gévry et al. (2004). They used an *Npc1*−/− female model, and submitted them to a transplant of a kidney capsule from wild types. Females had their ovulation and formation of corpora lutea restored following this intervention. Gonadotropin treatment induced ovulation and restored the expression of steroidogenic proteins. Additionally, the chronic treatment of knockout females with 17-β-estradiol restored the volume of the pituitary gland, as well as prolactin expression and folliculogenesis. Thus, they also concluded that NPC1 interferes in the hypothalamic-pituitary-ovarian feedback loop and consequently affects estrogen production (Gévry et al., 2004). The same group detected the importance of this protein in respect of adrenal development and function (Gévry and Murphy 2002).

Busso et al. (2010) examined NPC2 knockout females. The NPC1 and NPC2 proteins function cooperatively to catalyze cholesterol efflux from lysosomes. It is known that NPC1 is expressed in ovarian cells and female NPC1 deficient mice are infertile. In this study, the authors evaluated the location of NPC2 in the female reproductive tract. Ovarian NPC2 was present in theca and luteal cells, which use cholesterol to produce estradiol and progesterone, respectively. *Npc2*−/− female mice had altered estrous cycles, were infertile, with normal folliculogenesis only until antral stage, but no formation of the luteal body. Serum estradiol was reduced and ovarian cholesterol was stored in knockout mice, suggesting a defect in cholesterol export from intracellular stores. The authors demonstrated that NPC2 played a role in the traffic of ovarian cholesterol which is required for steroid synthesis and to support follicle maturation, ovulation and luteinization.

Fan et al. (2006) evaluated the same *Npc1−/−* mice and detected morphological sperm defects, low sperm number in their epididymal cauda and that gametes did not interact efficiently with the pellucid zone of oocytes from wild-type females.

Xie et al. (2006) used *Npc1*−/− mice to evaluate three sources for the capture of cellular cholesterol: LDL derived cholesterol, *de novo* synthesis and scavenger receptor mediated uptake of HDL cholesteryl ester. The rates of net cholesterol acquisition by these three pathways were measured in the adrenal, ovary, and testes. Plasma concentrations of testosterone, progesterone and corticosterone were similar to controls or even elevated in *Npc1*−/− mice. Thus, according to the authors, the impairment of cholesterol acquisition through the NPC1-dependent, clathrin-coated pit pathway did not limit the availability of cholesterol substrate for steroid hormone synthesis in the steroidogenic cells. However, despite normal hormone conditions, significant evidence of reproductive damage was reported in many studies as a result of the unsuitable lipid storage in the cellular environment.

Roff et al. (1993) evaluated *Npc1*−/− mice and detected lower intratesticular testosterone production, small seminal vesicles, changes in seminiferous tubules and in Leydig cells. Hepatic sterol carrier proteins were studied by Roff et al. (1992) and they detected that the expression of these proteins varies according to sexual development. Their expression in male and female *Npc1*−/− mice declined during sexual maturing.

Wang et al. (2019) evaluated a model of mucolipidosis Type IV, caused by a deficiency of a lysosomal ion channel and not directly by a lysosomal hydrolase. The absence of the ionic channel interferes in lysosomal pH and 5-month-old females are completely unfertile. Progesterone deficiency was detected 4.5 days after the post coitum/gestation day. Histological analysis showed less defined corpus luteal, extensive luteal cell vacuolization and degeneration. This study demonstrated a novel function of this lysosomal ion channel in maintaining luteal cell integrity and function.

### Glycoproteinoses

Taylor et al. (1989) evaluated dogs with fucosidosis, an LSD caused by genetic mutation of α-L-fucosidase. The enzymatic inactivity results in storage of glycoasparagines with terminal fucose residues in neurons and visceral tissues. The mutation in dogs, which is spontaneous, was associated with a range of significant reproductive damage, including to vacuoles in the testes and epididymis, reduced sperm number, abnormal sperm morphology and motility and altered surface glycoprotein composition during epididymal transit. Similar results were found by Veeramachaneni et al. (1998), who reported important spermatic damages and malformation of acrosomes.

Hu et al. (2012) studied a model of galactosialidosis, caused by the deficiency of serine carboxypeptidase protective protein/cathepsin A (PPCA). PPCA forms a complex with neuraminidase 1 (NEU1) and β-galactosidase (β-GAL). Patients with galactosialidosis develop a complete deficiency of NEU1 and a partial deficiency of β–GAL. Animals in male and female models are known to be infertile but after being injected with recombinant retroviral vectors expressing PPCA, their expression of PPCA and NEU1 recovered in reproductive tissues (testis, epididymis, ovary and uterus) and their fertility was restored.

### Other LSDs

Geist and Lüllmann-Rauch (1994) induced lipidosis with specific drugs and evaluated the effect on the estrous cycle and on the vaginal and uterine epithelia. After 2 weeks of continuous administration of chlorphentermine and imipramine, both drugs that interfere in lipid degradation, uterine and vaginal epithelia were vacuolated under ultrastructural examination and estrous cycle were stagnant as the lipidic storage interfered in steroidogenesis and all target organs and glands.

Ponce et al. (1999) observed the expression of acid glucosidase (GAA) in different tissues throughout mouse development. The expression of GAA was higher in Sertoli cells in comparison to other cell types. GAA is a lysosomal enzyme that cleaves glycogen and its absence results in Pompe disease.

### Studies Related to Lysosomal Proteins Important to Reproduction

Some studies did not use a specific LSD model, but explored the influence of lysosomal enzymes on germ cells and reproductive tissues. Hermo and Andonian (2003) evaluated two lysosomal enzymes: cathepsin D and sulphated glycoprotein 1 (SGP) in the epididymis. Their findings reinforced the importance of both hydrolases on endocytosis throughout the epididymal ducts in respect of sperm maturation. Another study reported that the activity of β-hexosaminidase is many times higher in the epididymis than in other tissues. The enzyme was detected in Sertoli cells, in the testicular interstitium and in epithelial cells of the epididymis (Hermo et al., 1997), evidencing the importance of this lysosomal hydrolase to a coordinated male germ cell production and maturing.

## Conclusion

Studies involving LSD models commonly investigate the muscles, bones, brain, heart, kidneys, liver and spleen because damage is more evident in these organs. Reproductive organs and tissue are poorly explored. The available pre-clinical studies showed us clear evidences that sperm, oocytes, testis, epididymis and even accessory glands may be affected, especially in sphingolipidoses and lipidoses. Some studies revealed only subtle changes in reproductive parameters, but given the fact that rodents are extremely fertile, any sign of subfertility must be taken seriously. LSDs are progressive diseases and some of them can be treated. Thus, we suggest that the development of children and teenagers must be monitored by physicians considering the time point of disease progression and the possible implications for reproduction and any possible treatments. Adults should also be monitored in this respect, especially those who are planning to have children. Moreover, it is important to publish case studies related to this area.

## References

[B1] Adamali H. I. Somani I. H. Huang J. Q. Gravel R. A. Trasler J. M. Hermo L. (1999b). II. Characterization and Development of the Regional- and Cellular-specific Abnormalities in the Epididymis of Mice with Beta-Hexosaminidase A Deficiency. J. Androl. 20 (6), 803–824. 10591619

[B2] Adamali H. I. Somani I. H. Huang J. Q. Mahuran D. Gravel R. A. Trasler J. M. (1999a). I. Abnormalities in Cells of the Testis, Efferent Ducts, and Epididymis in Juvenile and Adult Mice with Beta-Hexosaminidase A and B Deficiency. J. Androl. 20 (6), 779–802. 10591618

[B3] Anbu A. T. Mercer J. Wraith J. E. (2006). Effect of Discontinuing of Laronidase in a Patient with Mucopolysaccharidosis Type I. J. Inherit. Metab. Dis. 29 (1), 230–231. 10.1007/s10545-006-0237-8 16601901

[B6] Bacchus H. Peterson D. I. (1980). Pregnancy Complicated by Myelopathy Due to Maroteaux-Lamy Syndrome. Am. J. Obstet. Gynecol. 15136 (2), 259–260. 10.1016/0002-9378(80)90610-9 6766276

[B78] Barbosa Mendes A. do Nascimento C. C. D'Almeida V. (2019). Sexual Behaviour in a Murine Model of Mucopolysaccharidosis Type I (MPS I). PLoS One 14 (12), e0220429. 10.1371/journal.pone.0220429 31834922 PMC6910675

[B8] Busso D. Oñate-Alvarado M. J. Balboa E. Castro J. Lizama C. Morales G. (2014). Spermatozoa from Mice Deficient in Niemann-Pick Disease Type C2 (NPC2) Protein Have Defective Cholesterol Content and Reduced *In Vitro* Fertilising Ability. Reprod. Fertil. Dev. 26 (4), 609–621. 10.1071/RD12059 24709320

[B9] Busso D. Oñate-Alvarado M. J. Balboa E. Zanlungo S. Moreno R. D. (2010). Female Infertility Due to Anovulation and Defective Steroidogenesis in NPC2 Deficient Mice. Mol. Cell Endocrinol. 315 (1-2), 299–307. 10.1016/j.mce.2009.10.011 19883728

[B10] Butler A. Gordon R. E. Gatt S. Schuchman E. H. (2007). Sperm Abnormalities in Heterozygous Acid Sphingomyelinase Knockout Mice Reveal a Novel Approach for the Prevention of Genetic Diseases. Am. J. Pathol. 170 (6), 2077–2088. 10.2353/ajpath.2007.061002 17525274 PMC1899442

[B11] Butler A. He X. Gordon R. E. Wu H.-S. Gatt S. Schuchman E. H. (2002). Reproductive Pathology and Sperm Physiology in Acid Sphingomyelinase-Deficient Mice. Am. J. Pathol. 161 (3), 1061–1075. 10.1016/S0002-9440(10)64267-8 12213735 PMC1867239

[B12] Castorina M. Antuzzi D. Richards S. M. Cox G. F. Xue Y. (2015). Successful Pregnancy and Breastfeeding in a Woman with Mucopolysaccharidosis Type I while Receiving Laronidase Enzyme Replacement. Therapy. Clin. Exp. Obstet. Gynecol. 42 (1), 108–113. 25864295

[B13] Chung S. Ma X. Liu Y. Lee D. Tittiger M. Ponder K. P. (2007). Effect of Neonatal Administration of a Retroviral Vector Expressing α-l-iduronidase upon Lysosomal Storage in Brain and Other Organs in Mucopolysaccharidosis I Mice. Mol. Genet. Metab. 90 (2), 181–192. 10.1016/j.ymgme.2006.08.001 16979922

[B14] Delgado C. Kent C. Sedensky M. Ciliberto C. Landau R. (2015). Management of Labor and Delivery in a Woman with Morquio Syndrome. Int. J. Obstet. Anesth. 24 (4), 383–387. 10.1016/j.ijoa.2015.08.015 26431780

[B15] do Nascimento C. C. Junior O. A. D'Almeida V. (2014). Analysis of Male Reproductive Parameters in a Murine Model of Mucopolysaccharidosis Type I (MPS I). Int. J. Clin. Exp. Pathol. 157 (6), 3488–3497. PMC4097293 25031781

[B16] do Nascimento C. C. Aguiar O. Viana G. M. D'Almeida V. (2019a). Evidence that Glycosaminoglycan Storage and Collagen Deposition in the Cauda Epididymidis Does Not Impair Sperm Viability in the Mucopolysaccharidosis Type I Mouse Model. Reprod. Fertil. Dev. 32 (3), 304–312. 10.1071/RD19144 31679559

[B17] do Nascimento C. C. Aguiar O. Viana G. M. D’Almeida V. (2020). Morphological Damage in Sertoli, Myoid and Interstitial Cells in a Mouse Model of Mucopolysaccharidosis Type I (MPS I). Mol. Biol. Rep. 48 (1), 363–370. 10.1007/s11033-020-06055-5 33319323

[B18] do Nascimento C. C. Junior O. A. D’Almeida V. (2019b). Morphologic Description of Male Reproductive Accessory Glands in a Mouse Model of Mucopolysaccharidosis Type I (MPS I). J. Mol. Hist. 51 (2), 137–145. 10.1007/s10735-020-09864-x 32162173

[B19] Donohue C. Marion S. Erickson R. P. (2009). Expression ofNpc1 in Glial Cells Corrects Sterility inNpc1 -/- Mice. J. Appl. Genet. 50, 385–390. 10.1007/BF03195698 19875890

[B20] Erickson R. P. Kiela M. Devine P. J. Hoyer P. B. Heidenreich R. A. (2002). Mdr1a Deficiency Corrects Sterility in Niemann-Pick C1 Protein Deficient Female Mice. Mol. Reprod. Dev. 62 (2), 167–173. 10.1002/mrd.10093 11984826

[B21] Fan J. Akabane H. Graham S. N. Richardson L. L. Zhu G.-Z. (2006). Sperm Defects in Mice Lacking a Functional Niemann-Pick C1 Protein. Mol. Reprod. Dev. 73 (10), 1284–1291. 10.1002/mrd.20559 16850391

[B22] Geist S. H. Lüllmann-Rauch R. (1994). Experimentally Induced Lipidosis in Uterine and Vaginal Epithelium of Rats. Ann. Anat. - Anatomischer Anzeiger 176 (1), 3–9. 10.1016/s0940-9602(11)80404-8 8304588

[B23] Gévry N. Y. Lopes F. L. Ledoux S. Murphy B. D. (2004). Aberrant Intracellular Cholesterol Transport Disrupts Pituitary and Ovarian Function. Mol. Endocrinol. 18 (7), 1778–1786. 10.1210/me.2003-0323 15105438

[B24] Gévry N. Y. Murphy B. D. (2002). The Role and Regulation of the Niemann-Pick C1 Gene in Adrenal Steroidogenesis. Endocr. Res. 28 (4), 403–412. 10.1081/erc-120016815 12530642

[B25] Hauser A. C. Gessl A. Harm F. Wiesholzer M. Kleinert J. Wallner M. (2005). Hormonal Profile and Fertility in Patients with Anderson-Fabry Disease. Int. J. Clin. Pract. 59 (9), 1025–1028. 10.1111/j.1742-1241.2005.00620.x 16115176

[B26] Hermo L. Adamali H. I. Mahuran D. Gravel R. A. Trasler J. M. (1997). β-Hexosaminidase Immunolocalization and α- and β-subunit Gene Expression in the Rat Testis and Epididymis. Mol. Reprod. Dev. 4646 (3), 227–242. 10.1002/(sici)1098-2795(199703)46:3<227:aid-mrd1>3.0.co;2-r 9041125

[B27] Hermo L. Andonian S. (2003). Regulation of Sulfated Glycoprotein-1 and Cathepsin D Expression in Adult Rat Epididymis. J. Androl. 24 (3), 408–422. 10.1002/j.1939-4640.2003.tb02690.x 12721218

[B28] Higuchi T. Shimizu H. Fukuda T. Kawagoe S. Matsumoto J. Shimada Y. (2012). Enzyme Replacement Therapy (ERT) Procedure for Mucopolysaccharidosis Type II (MPS II) by Intraventricular Administration (IVA) in Murine MPS II. Mol. Genet. Metab. 107 (1-2), 122–128. 10.1016/j.ymgme.2012.05.005 22704483

[B29] Hu H. Gomero E. Bonten E. Gray J. T. Allay J. Wu Y. (2012). Preclinical Dose-Finding Study with a Liver-Tropic, Recombinant AAV-2/8 Vector in the Mouse Model of Galactosialidosis. Mol. Ther. 20 (2), 267–274. 10.1038/mt.2011.227 22008912 PMC3277225

[B30] Hull E. M. Domingues J. M. Dominguez J. M. (2007). Sexual Behavior in Male Rodents. Horm. Behav. 52 (1), 45–55. 10.1016/j.yhbeh.2007.03.030 17499249 PMC1952538

[B31] Juneja S. C. (2002). Development of Infertility at Young Adult Age in a Mouse Model of Human Sandhoff Disease. Reprod. Fertil. Dev. 14 (7-8), 407–412. 10.1071/rd02060 12617783

[B32] Kongmanas K. Saewu A. Kiattiburut W. Baker M. A. Faull K. F. Burger D. (2021). Accumulation of Seminolipid in Sertoli Cells Is Associated with Increased Levels of Reactive Oxygen Species and Male Subfertility: Studies in Aging Arsa Null Male Mice. Antioxidants 10 (6), 912. 10.3390/antiox10060912 34199863 PMC8227610

[B33] Körschen H. G. Yildiz Y. Raju D. N. Schonauer S. Bönigk W. Jansen V. (2013). The Non-lysosomal β-Glucosidase GBA2 Is a Non-integral Membrane-Associated Protein at the Endoplasmic Reticulum (ER) and Golgi. J. Biol. Chem. 288 (5), 3381–3393. 10.1074/jbc.M112.414714 23250757 PMC3561557

[B34] Levade T. Jaffrezou J. P. Jaffrézou J.-P. (1999). Signalling sphingomyelinases: which, where, how and why?11With the participation of Nathalie Andrieu-Abadie, Nathalie Augé, Bruno Ségui, Emmanuelle Uro-Coste, Robert Salvayre, INSERM Unit 466, Laboratoire de Biochimie, Maladies Métaboliques, Institut Louis Bugnard, Bât. L3, C.H.U. Rangueil, 1 Avenue Jean Poulhès, E 9910 Toulouse Cedex 4, France, and Christine Bezombes, Véronique Mansat-De Mas, INSERM CJF 9503, Institut Claudius Régaud, Toulouse, France. Biochim. Biophys. Acta (Bba) - Mol. Cel Biol. Lipids 191438 (1), 1–17. 10.1016/s1388-1981(99)00038-4

[B35] Luddi A. Strazza M. Carbone M. Moretti E. Costantinoceccarini E. (2005). Galactosylceramidase Deficiency Causes Sperm Abnormalities in the Mouse Model of Globoid Cell Leukodystrophy. Exp. Cel Res. 10304 (1), 59–68. 10.1016/j.yexcr.2004.10.034 15707574

[B36] Marques A. R. A. Saftig P. (2019). Lysosomal Storage Disorders - Challenges, Concepts and Avenues for Therapy: beyond Rare Diseases. J. Cel Sci 16132 (2), jcs221739. 10.1242/jcs.221739 30651381

[B37] McGlynn R. Dobrenis K. Walkley S. U. (2004). Differential Subcellular Localization of Cholesterol, Gangliosides, and Glycosaminoglycans in Murine Models of Mucopolysaccharide Storage Disorders. J. Comp. Neurol. 20480 (4), 415–426. 10.1002/cne.20355 15558784

[B38] Medina D. L. Ballabio A. (2015). Lysosomal Calcium Regulates Autophagy. Autophagy 11 (6), 970–971. 10.1080/15548627.2015.1047130 26000950 PMC4502748

[B77] Milazzo J. P. Bironneau A. Vannier J. P. Liard-Zmuda F. Kanold A. Macé B. (2014). Precocious Initiation of Spermatogenesis in a 19-month-old boy with Hurler Syndrome. Basic Clin Androl. 1 (24), 8. 10.1186/2051-4190-24-8 PMC4349721 25780582

[B39] Morales C. R. Zhao Q. El-Alfy M. Suzuki K. (2000a). Targeted Disruption of the Mouse Prosaposin Gene Affects the Development of the Prostate Gland and Other Male Reproductive Organs. J. Androl. 21 (6), 765–775. 10.1002/j.1939-4640.2000.tb03407.x 11105903

[B40] Morales C. R. Zhao Q. Lefranco S. Heim D. (2000b). Role of Prosaposin in the Male Reproductive System: Effect of Prosaposin Inactivation on the Testis, Epididymis, Prostate, and Seminal Vesicles. Arch. Androl. 44 (3), 173–186. 10.1080/014850100262146 10864364

[B41] Murray G. J. Anver M. R. Kennedy M. A. Quirk J. M. Schiffmann R. (2007). Cellular and Tissue Distribution of Intravenously Administered Agalsidase Alfa. Mol. Genet. Metab. 90 (3), 307–312. 10.1016/j.ymgme.2006.11.008 17188539 PMC1839873

[B42] Papaxanthos-Roche A. Maillard A. Chansel-Debordeaux L. Albert M. Patrat C. Lidove O. (2019). Semen and Male Genital Tract Characteristics of Patients with Fabry Disease: the FERTIFABRY Multicentre Observational Study. Basic Clin. Androl. 29, 297. 10.1186/s12610-019-0088-4 PMC6518714 31123589

[B43] Parenti G. Andria G. Ballabio A. (2015). Lysosomal Storage Diseases: from Pathophysiology to Therapy. Annu. Rev. Med. 66, 471–486. 10.1146/annurev-med-122313-085916 25587658

[B44] Parenti G. Pignata C. Vajro P. Salerno M. (2013). New Strategies for the Treatment of Lysosomal Storage Diseases (Review). Int. J. Mol. Med. 31 (1), 11–20. 10.3892/ijmm.2012.1187 23165354

[B75] Parkinson-Lawrence E. J. Shandala T. Prodoehl M. Plew R. Borlace G. N. Brooks D. A. (2010). Lysosomal Storage Disease: Revealing Lysosomal Function and Physiology. Physiology 25 (2), 105–15. 10.1152/physiol.00041.2009 20430954

[B45] Piomboni P. Governini L. Gori M. Puggioni E. Costantino-Ceccarini E. Luddi A. (2014). New Players in the Infertility of a Mouse Model of Lysosomal Storage Disease: The Hypothalamus-Pituitary-Gonadal Axis. Front. Endocrinol. 64, 204. 10.3389/fendo.2013.00204 PMC3880942 24432014

[B46] Platt F. M. d’Azzo A. Davidson B. L. Neufeld E. F. Tifft C. J. (2018). Lysosomal Storage diseasesErratum in: Nat Rev Dis Primers, 18; 4(1Erratum in. Nat. Rev. Dis. Primersnat Rev. Dis. Primers 4 (11), 273634. 10.1038/s41572-018-0025-4

[B47] Ponce E. Witte D. P. Hirschhorn R. Huie M. L. Grabowski G. A. (1999). Murine Acid α-Glucosidase. Am. J. Pathol. 154 (4), 1089–1096. 10.1016/s0002-9440(10)65361-8 10233847 PMC1866561

[B76] Remérand G. Merlin E. Froissart R. Brugnon F. Kanold J. Janny L. (2009). Four Successful Pregnancies in a Patient with Mucopolysaccharidosis Type i Treated by Allogeneic Bone Marrow Transplantation. J. Inherit. Metab. Dis. 32 , 111–3. 10.1007/s10545-009-1095-y 19280364

[B48] Roff C. F. Pastuszyn A. Strauss J. F. III Billheimer J. T. Vanier M. T. Brady R. O. (1992). Deficiencies in Sex-Regulated Expression and Levels of Two Hepatic Sterol Carrier Proteins in a Murine Model of Niemann-Pick Type C Disease. J. Biol. Chem. 5267 (22), 15902–15908. 10.1016/s0021-9258(19)49619-1 1639819

[B49] Roff C. F. Strauss J. F. III Goldin E. Jaffe H. Patterson M. C. Agritellis G. C. (1993). The Murine Niemann-Pick Type C Lesion Affects Testosterone Production. Endocrinology 1133 (6), 2913–2923. 10.1210/endo.133.6.8243319 8243319

[B50] Saudubray J.-M. Garcia-Cazorla À. (2018). Inborn Errors of Metabolism Overview. Pediatr. Clin. North America 65 (2), 179–208. 10.1016/j.pcl.2017.11.002 29502909

[B51] Schneider A. P. Matte U. Pasqualim G. Tavares A. M. V. Mayer F. Q. Martinelli B. (2016). Deleterious Effects of Interruption Followed by Reintroduction of Enzyme Replacement Therapy on a Lysosomal Storage Disorder. Translational Res. 176, 29–37. 10.1016/j.trsl.2016.05.002 27450046

[B53] Scriver C. R. Beaudet A. L. Sly W. S. Valle D. Childs B. Kinzler K. W. (2001). The Metabolic and Molecular Bases of Inherited Disease. 8th ed. New York: McGraw-Hill.

[B54] Sechi A. Deroma L. Lapolla A. Paci S. Melis D. Burlina A. (2013). Fertility and Pregnancy in Women Affected by Glycogen Storage Disease Type I, Results of a Multicenter Italian Study. J. Inherit. Metab. Dis. 36 (1), 83–89. 10.1007/s10545-012-9490-1 22562700

[B55] Seyrantepe V. Demir S. A. Timur Z. K. Von Gerichten J. Marsching C. Erdemli E. (2018). Murine Sialidase Neu3 Facilitates GM2 Degradation and Bypass in Mouse Model of Tay-Sachs Disease. Exp. Neurol. 299 (Pt A), 26–41. 10.1016/j.expneurol.2017.09.012 28974375

[B56] Shen J.-S. Meng X.-L. Wight-Carter M. Day T. S. Goetsch S. C. Forni S. (2015). Blocking Hyperactive Androgen Receptor Signaling Ameliorates Cardiac and Renal Hypertrophy in Fabry Mice. Hum. Mol. Genet. 124 (11), 3181–3191. 10.1093/hmg/ddv070 25701874

[B57] Soper B. W. Pung A. W. Vogler C. A. Grubb J. H. Sly W. S. Barker J. E. (1999). Enzyme Replacement Therapy Improves Reproductive Performance in Mucopolysaccharidosis Type VII Mice but Does Not Prevent Postnatal Losses. Pediatr. Res. 45, 180–186. 10.1203/00006450-199902000-00004 10022587

[B58] Staropoli J. F. Haliw L. Biswas S. Garrett L. Hölter S. M. Becker L. (2012). Large-Scale Phenotyping of an Accurate Genetic Mouse Model of JNCL Identifies Novel Early Pathology outside the Central Nervous System. PLoS One 7 (6), e38310. 10.1371/journal.pone.0038310 22701626 PMC3368842

[B59] Stewart F. Bentley A. Burton B. K. Guffon N. Hale S. L. Harmatz P. R. (2016). Expert Opinions on Managing Fertility and Pregnancy in Patients with Mucopolysaccharidosis. J. Inborn Errors Metab. Screen. 4, 232640981666937. 10.1177/2326409816669375

[B60] Stinchi S. Lüllmann-Rauch R. Hartmann D. Coenen R. Beccari T. Orlacchio A. (1999). Targeted Disruption of the Lysosomal -Mannosidase Gene Results in Mice Resembling a Mild Form of Human -Mannosidosis. Hum. Mol. Genet. 8 (8), 1365–1372. 10.1093/hmg/8.8.1365 10400983

[B61] Suzuki K. Ezoe T. Tohyama J. Matsuda J. Vanier M. Suzuki K. (2003). Are Animal Models Useful for Understanding the Pathophysiology of Lysosomal Storage Disease. Acta Paediatr. Suppl. 92 (443), 54–62. 10.1111/j.1651-2227.2003.tb00223.x 14989467

[B62] Tanphaichitr N. Kongmanas K. Faull K. F. Whitelegge J. Compostella F. Goto-Inoue N. (2018). Properties, Metabolism and Roles of Sulfogalactosylglycerolipid in Male Reproduction. Prog. Lipid Res. 72, 18–41. 10.1016/j.plipres.2018.08.002 30149090 PMC6239905

[B63] Taylor R. M. Martin I. C. A. Farrow B. R. H. (1989). Reproductive Abnormalities in Canine Fucosidosis. J. Comp. Pathol. 100 (4), 369–380. 10.1016/0021-9975(89)90002-9 2760271

[B64] Trasler J. Saberi F. Somani I. H. Adamali H. I. Huang J.-Q. Fortunato S. R. (1998). Characterization of the Testis and Epididymis in Mouse Models of Human Tay Sachs and Sandhoff Diseases and Partial Determination of Accumulated Gangliosides*. Endocrinology 139 (7), 3280–3288. 10.1210/endo.139.7.6117 9645704

[B65] Veeramachaneni D. N. Smith M. O. Ellinwood N. M. (1998). Deficiency of Fucosidase Results in Acrosomal Dysgenesis and Impaired Sperm Maturation. J. Androl. 19 (4), 444–449. 9733147

[B66] Walkley S. (2004). Secondary Accumulation of Gangliosides in Lysosomal Storage Disorders. Semin. Cel Develop. Biol. 15 (4), 433–444. 10.1016/j.semcdb.2004.03.002 15207833

[B67] Walkley S. U. Thrall M. A. Haskins M. E. Mitchell T. W. Wenger D. A. Brown D. E. (2005). Abnormal Neuronal Metabolism and Storage in Mucopolysaccharidosis Type VI (Maroteaux-Lamy) Disease. Neuropathol. Appl. Neurobiol. 31 (5), 536–544. 10.1111/j.1365-2990.2005.00675.x 16150124

[B68] Wang Z. El Zowalaty A. E. Li Y. Andersen C. L. Ye X. (2019). Association of Luteal Cell Degeneration and Progesterone Deficiency with Lysosomal Storage Disorder Mucolipidosis Type IV in Mcoln1−/− Mouse Model†. Bio of Repro 101 (4), 782–790. 10.1093/biolre/ioz126 PMC6864120 31317194

[B69] Wilson A. Lavery C. Stewart F. Thomas S. Cavell D. Brandon R. (2018). Mucopolysaccharidosis and Adulthood: Genetics, Inheritance, and Reproductive Options. J. Child. Sci. 8 1, 138–143. 10.1055/s-0038-1667347

[B70] Wu A. Anupriwan A. Iamsaard S. Chakrabandhu K. Santos D. C. Rupar T. (2007). Sperm Surface Arylsulfatase A Can Disperse the Cumulus Matrix of Cumulus Oocyte Complexes. J. Cel. Physiol. 213 (1), 201–211. 10.1002/jcp.21113 17474085

[B71] Xie C. Richardson J. A. Turley S. D. Dietschy J. M. (2006). Cholesterol Substrate Pools and Steroid Hormone Levels Are normal in the Face of Mutational Inactivation of NPC1 Protein. J. Lipid Res. 47 (5), 953–963. 10.1194/jlr.M500534-JLR200 16461760

[B72] Xu H. Kongmanas K. Kadunganattil S. Smith C. E. Rupar T. Goto-Inoue N. (2011). Arylsulfatase A Deficiency Causes Seminolipid Accumulation and a Lysosomal Storage Disorder in Sertoli Cells. J. Lipid Res. 52, 2187–2197. 10.1194/jlr.M019661 21965315 PMC3220287

[B73] Yildiz Y. Matern H. Thompson B. Allegood J. C. Warren R. L. Ramirez D. M. O. (2006). Mutation of β-glucosidase 2 Causes Glycolipid Storage Disease and Impaired Male Fertility. J. Clin. Invest. 116 (11), 2985–2994. 10.1172/JCI29224 17080196 PMC1626112

[B74] Zhu M. Lovell K. L. Patterson J. S. Saunders T. L. Hughes E. D. Friderici K. H. (2006). β-Mannosidosis Mice: a Model for the Human Lysosomal Storage Disease. Hum. Mol. Genet. 15 (3), 493–500. 10.1093/hmg/ddi465 16377659

